# 

**Published:** 1858-03

**Authors:** 


*American Medical Association.*—The Eleventh Annual Meeting of this Association, will be held in the city of Washington, on Tuesday, May 4th, 1858. The secretaries of all societies and other bodies entitled to representation in the Association, are requested to forward to the secretary, Alexander I. Semmes, Washington, correct lists of their delegations as soon as they may be appointed; and the Committee of Arrangements earnestly desire that the appointments may be made at as early a period as possible.—*The Med. News and Library.*

---

*New Jourual of Physiology.*—Dr. Brown Sequard has sent us the prospectus of a journal of physiology, of which he is to be the principal editor, and which is to be published quarterly, on the 1st of January, April, July, and October—each number to contain from one hundred and sixty to two hundred pages. He is to be assisted in his enterprise by Drs. Ch. Robin, Ch. Rouget, and Tholozan, &c.

His journal, in addition to pure physiology, will embrace:

1. Organic chemistry, hygiene, toxicology, and legal medicines in their relations to physiology.

2. Descriptive anatomy, comparative anatomy, teratology, and normal and pathological histology, so far as they illustrate physiology.

3. The applications of physiology to the practice of medicine, surgery, and obstetrics.

The subscription will be 18 francs, at Paris, payable in advance.

We invite the attention of the profession in this country to this Journal. It may be obtained through I. Pennington & Son, of Philadelphia.—*Ibid.*

